# Someone else's ears: Metacognitive auditory perspective taking in young and older adults

**DOI:** 10.1177/20416695251349735

**Published:** 2025-07-09

**Authors:** Chiara Valzolgher, Elena Gessa, Elena Giovanelli, Francesco Pavani

**Affiliations:** 1 Center for Mind/Brain Sciences (CIMeC), University of Trento, Italy; 2 Centro Interuniversitario di Ricerca “Cognizione, Linguaggio e Sordità” (CIRCLeS), Trento, Italy

**Keywords:** metacognition, aging, perspective taking, hearing in noise

## Abstract

Understanding others’ listening experiences is an instance of social metacognition. We investigate attributed listening experiences to fictional others in two online experiments involving two groups, young and older adults with normal hearing. We assessed the similarity between the judgement they gave for themselves and for two fictional characters (same or different age), with respect to listening experiences and effort required across various listening scenarios. In Exp. 1, all characters were described as having normal hearing; in Exp. 2, we introduced one additional older character with hearing loss. In both experiments, younger adults judged the older characters as experiencing more effort, irrespective of hearing loss. Instead, older adults reported to experience less effort than older characters, irrespective of characters’ hearing status and judged themselves closer to the young character. These findings show disparities in metacognitive auditory perspective taking in young and older adults, documenting a potential self-serving bias of older adults.

## How to cite this article

Valzolgher, C., Gessa, E., Giovanelli, E., & Pavani, F. (2025). Someone else's ears: Metacognitive auditory perspective taking in young and older adults. *i-Perception*, 16(4), 1–18. https://doi.org/10.1177/20416695251349735

People typically listen while immersed in a variety of different acoustic scenarios. Their auditory experience requires both to understand speech in quiet and in the presence of competing sounds. This task entails efficient encoding of acoustic information from the external environment and the ability to attribute meaning to them (Shinn-Cunningham, 2008; Sussman, 2017). In addition, the subjective experience associated with this task involves the ability to reflect on one's own perceptive skills, evaluate the reliability of received information, and recognise environments that are more or less favourable for listening (Kamerer et al., 2022). This ability to describe subjective feelings about one's own listening experience is an instance of *metacognition*, the high-level capacity to reflect on one's own way of perceiving, manipulating information, and thinking (Flavell, 1979; Fleming, 2024). Metacognition comprises knowledge and beliefs about one's own abilities (i.e., metacognitive knowledge) and monitoring of the ongoing cognitive processes (i.e., metacognitive monitoring; Ackerman & Thompson, 2017). Considering listening abilities, if people have good metacognitive knowledge, they should be capable of accurately representing their own listening skills. From now on, we refer to these abilities as ‘listening metacognition’.

Studies investigating listening experiences have often taken for granted this ability by assuming that people are able to represent their listening ability in different scenarios. An example is the speech, spatial, and qualities of hearing questionnaire, that is, the SSQ (e.g., in Gatehouse & Noble, 2004; Singh et al., 2010), in which people are asked to rate their difficulties in different listening scenarios. While the SSQ does not directly assess listening metacognition, answers obtained from its administration showed that people are able to discern hearing difficulties based on the scenarios (e.g., hearing in quiet or noisy environments). This finding could in part suggest that people are effectively aware about the impact of external factors on their hearing experience, reflecting good listening metacognition. However, while these results showed that people can evaluate their own listening abilities, little is known about how people evaluate *others’ listening experiences* in everyday life.

The representation of others’ listening experiences relies on the ability to assume others’ perspectives (i.e., perspective taking). Perspective taking has been defined as the capacity to imagine the mental and emotional states of other people, standing between empathy-related phenomena (Bertrand et al., 2018; Frith & Frith, 2005). This capacity has a strong relationship with metacognitive abilities as they shared part of neural circuits (Vaccaro & Fleming, 2018) as well as the same development period (Hembacher & Ghetti, 2014). The ability to take the point of view of others has been studied historically using perceptual tasks related to vision. For instance, in the classical three-mountain task (Piaget & Inhelder, 1956), participants are placed in front of a 3D-model of a landscape and they are asked to report what others can observe from different angles. To solve the task, it is crucial to take other spatial-visual points of view (Flavell, 2004) or at least to code objects assuming an allocentric perspective (Newcombe, 1989).

This task focused on visual perspective taking, but to the best of our knowledge perspective taking in the domain of acoustic perception has yet to be defined. Consider for instance a face-to-face conversation between a young and an older adult: imagine they are in a restaurant and with a disturbing background noise. While for the young individual this conversation could be challenging, for the older adult it could be extremely frustrating. Assuming the acoustic point of view of the older adult by the young one becomes fundamental in order to imagine acoustic challenges he/she is experiencing at that moment. Crucially, having access to this information can consequently allow the young adult to adapt his/her behaviour to facilitate the conversation or choose to change location. From now on we will refer to this notion as interpersonal listening metacognition.

How can we create a representation of the others’ acoustic experience? One possibility is that we rely on the expectation that a person similar to us will perceive the auditory environment similarly to how we perceive it. In addition, we could rely on a priori knowledge we have available about the others’ perceptual experience (De Lange et al., 2018; Otten et al., 2017). For instance, young adults could shape their interpersonal listening metacognition for older adults based on their knowledge of how aging affects auditory perception. Since age is known to be associated with cognitive and hearing declines, this general knowledge could allow us to have a mental representation of the potential listening difficulties experienced by older adults in a noisy environment (Banh et al., 2012; Ciorba et al., 2012; Glyde et al., 2011; Kuchinsky & Vaden, 2020; Pichora-Fuller, 1997). While addressing this issue could have substantial implications when aiming to promote the interactions across the lifespan, in typical and in clinical context, to the best of our knowledge, no study to date has considered the acoustic experience under the theoretical framework of perceptual perspective taking. In this study, we aimed to examine metacognitive perspective taking of sensory abilities within the domain of acoustic perception by focusing on the listening experience of two fictional characters of different ages.

In Experiment 1, we aimed to directly test the hypothesis that inferences regarding the listening experience of others are influenced by participants’ belonging to the same versus different age group. To this aim, we run an online study involving participants from two age groups, young and older adults, reporting no subjective hearing difficulties. We introduced them to fictional characters of the same or different age, namely a young individual named Marco (i.e., a common Italian name across all ages) and an older adult named Ezio. Both characters were described as having typical hearing for their age. We asked participants to rate their own perceived listening effort across various acoustic scenarios (Self-judgement), rate the perceived listening effort for the character of the same age group (e.g., Marco for young participants; Ingroup judgement), and rate the perceived listening effort for the character of the different age group (e.g., Ezio for young participants; Outgroup judgement). This questionnaire was modified from the SSQ scale, adapted here to refer to both self and others. In line with previous research on the SSQ scale, we kept the distinction between easy and difficult listening situations. Finally, to test to what extent participants relate to the Ingroup versus Outgroup fictional character in terms of perceptual experiences we implemented a second questionnaire. This was modified from the Inclusion of the Other in the Self (IOS) questionnaire, developed for the study of social closeness (Paladino et al., 2010; Schubert & Otten, 2002), here adapted for auditory experiences. If belonging to the same versus different age group impacts on the way we attribute listening experience to others, participants should perceive unknown others as having listening experience similar to themselves more for Ingroup than Outgroup characters.

## Experiment 1

### Materials and Method

#### Participants

Twenty-five young adults (age: 25.5 ± 2.8, 9 males) and 26 older adults (age: 70.5 ± 4.5, 12 males) took part in Experiment 1. All participants gave their informed consent before starting the experiment, which was conducted according to the criteria of the Declaration of Helsinki (1964, amended in 2013) and in accordance with the regulations at the University of Trento. We only included Italian native speakers and who did not report having hearing problems in a self-report questionnaire. Participants were recruited by using the official university channels, such as the mailing list and announcements on social media pages dedicated to experiments.

#### Stimuli

*The fictional characters.* Marco has been described by using the following words: ‘Marco is a 23-year-old boy, he studies at university and has normal vision and normal hearing’. Ezio has been described by using the following words: ‘Ezio is a 72-year-old individual, he is retired and has normal vision and normal hearing’.

**Figure 1.**
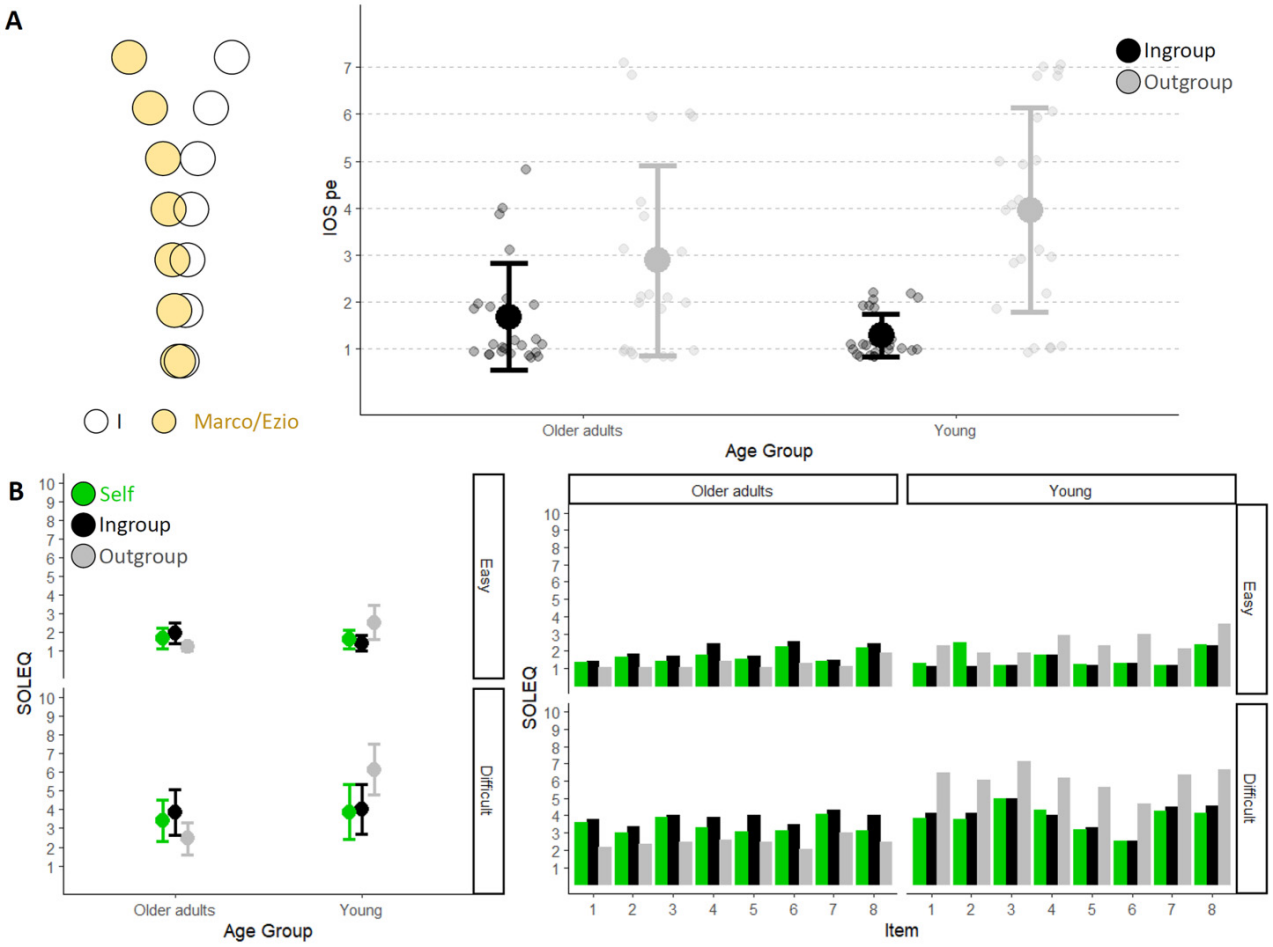
(A) On the left, an example of IOS-pe visualisation. Each circle is paired with a value solely for the purpose of selecting a response, which is then used for analysis: 1 corresponds to the most overlapping configuration, while 7 corresponds to the most distant configuration. On the right, IOS-pe mean values for young and older adults as a function of character of evaluation (Ingroup in black and Outgroup in grey). Older adults are on the left, while young adults are on the right. The error bars represent the standard deviation, and the smaller dots represent the response of each participant. (B) On the left, the mean response values provided by older adults (on the left) and young adults (on the right) as a function of the target of evaluation (Self in green, Ingroup in black and Outgroup in grey). The top graph refers to easy situations, while the bottom one refers to difficult situations. On the right, the mean values for each individual item (8 for each condition) are shown. Again, older adults are on the left, and young adults are on the right, with the easy condition on top and the difficult condition on the bottom. The colours vary depending on the target of evaluation (green for Self, black for Ingroup and grey for Outgroup). See Supplementary Information for average scores.

*IOS-pe (IOS questionnaire for perceptual experiences).* The questionnaire aimed to investigate to what extent the perceptual experiences with the fictional character were perceived as overlapping. To this aim, we adapted the Inclusion of the other in the self (IOS) questionnaire, adopted to investigate relationships in social sciences (e.g., Paladino et al., 2010; see Schubert & Otten, 2002). We asked participants to rate the similarity between their acoustic perceptual experience and that of the two characters we presented at the beginning of the experiment (i.e., Marco and Ezio). The instruction (originally presented in Italian) was ‘*Please select the figure that best describes your closeness to Marco from the point of view of perceptive/sensory experiences (e.g., acoustic perception…). When the two circles are very overlapping (Option 1) it means that you believe your perceptual experience is very similar to that of Marco (i.e., you believe that what you hear is no different from what Marco is able to hear). On the contrary, when the two circles are separated (Option 7), it means that you consider your perceptual experience different from that of Marco*’. Participants were required to choose one out of seven graphical representations of the relations between self and other experiences (Figure 1A).

*Self-Other Listening Experience Questionnaire (SOLEQ).* This questionnaire probed self and other perceived listening effort across different acoustic scenarios. To this aim, we developed a revised version of the SSQ scale in which questions are formulated both with respect to oneself and with respect to another person (i.e., the fictional character). We presented 16 different listening situations three times (one for subject: one's own experience, Marco's experience, and Ezio's experience) and asked participants to rate on a Likert scale spanning from 1 (low difficulty) to 10 (high difficulty) the amount of difficulty required by each situation assuming one's own perspective and the two characters’ perspectives. The 16 situations were divided into 8 easy (e.g., listening in a quiet place) and 8 more difficult experiences (e.g., listening in a noisy place; the original sentences in Italian and their translation in English are reported in the Supplementary Information). The items were presented completely randomised across conditions.

It is important to note that although the two characters presented are both male and were introduced with characteristics that define their life circumstances (e.g., studying and retired), participants were thoroughly instructed to focus on the listening experience of these two characters. Therefore, age appears to be the key factor distinguishing their perceptual experiences. Nonetheless, future research is needed to further explore how gender and different life circumstances shape our estimation of others’ listening experiences.

#### Procedure

The experiment was entirely conducted online by using Google Form. Participants were informed about the study and the instructions at the beginning of the form and we asked them to give their consent to take part in the study and fill in a form reporting their age, sex, mother-tongue, and self-assessed listening abilities. First, we briefly introduced the two characters (i.e., Marco and Ezio) and asked the participants to focus on their own perceptual experience and that of the characters presented. Then, participants were instructed to complete the *IOS-pe* and then the *SOLEQ.* At the end of the experiment, we asked them to tell us if they had received support from other people to perform the questionnaire. No one declared to be assisted. Participating required about 15 min.

#### Analysis

Data were collected through the administration of the IOS-pe and the SOLEQ. Regarding the IOS-pe analyses, raw data consisted of one value per condition per participant. For the SOLEQ, we had eight items per condition per participant. We considered all datasets in the analyses without averaging the data. In the case of figures and tables, we reported the means and the corresponding standard deviations. To analyse the SOLEQ data, we also computed an additional index: for each item, we calculated the absolute error difference between values reported for subjective versus others’ experiences. These differences represent the discrete amount of discrepancy between self and other experiences (Other-Self Discrepancy, OSD).

Since the dependent variables were ordinal, we used models that are suitable for this type of data, while also accounting for certain random effects in the analysis. This approach was particularly important for the SOLEQ data, as we included the item as a random effect in the model. For this analysis, we used R software in combination with RStudio, specifically R version 4.4.1, and employed the *clmm* function, which allowed us to handle ordinal responses that are not quantitative (Christensen, 2023). Note that we reported estimate values and z-values in absolute terms because we wanted to guide the reader towards the direction of the effect by directly referring to the actual data means. The means reported throughout the text and in the tables are the real means of the data, not the ones estimated by the models. Additionally, we explored the effects of the model using the *emmeans* function (Lenth, 2024), and applied the Tukey correction for multiple comparisons.

We also note that for the IOS-pe data, we had missing values for one participant, and therefore we removed them from the analyses related to this index (ID code is ‘Gucp’).

### Results

#### IOS-pe

Because our working hypothesis was that participants should rate the difficulties experienced by the Ingroup character as closer to their own compared to the experience of the Outgroup character, we first tested to what extent participants relate to the Ingroup versus Outgroup character in terms of perceptual experiences. To this end, we entered participants’ responses in the IOS-pe as dependent variables into a cumulative link model for ordinal variables with AGE (young, older adults) and CHARACTER of the EVALUATION (Ingroup, Outgroup) as fixed effect and participants as random effects. This analysis revealed that responses varied according to the character of the evaluation (Estimate = 4.22, *z* = 4.64, *p* < .001). Both groups of participants related more with the Ingroup compared to the Outgroup fictional character. However, the interaction effect was significant (Estimate = 2.21, *z* = 2.35, *p* = .02), indicating that the difference between the Ingroup and Outgroup was greater for young participants (Ingroup: 1.3 ± 0.5; Outgroup: 4.0 ± 2.2, Estimate = 4.22, *z* = 4.64, *p* < .001) than for older adults (Ingroup: 1.7 ± 1.1; Outgroup: 2.9 ± 2.0, Estimate = 2.01, *z* = 3.01, *p* = .01), who rated the Outgroup character (i.e., Marco) as closer to their subjective experience (Figure 1A).

#### SOLEQ

Having established that participants related more with the Ingroup than the Outgroup character, and that this difference was more prominent true for young participants, we turned to examine our main question concerning the ability to estimate the listening effort experienced by others in a given situation. First, we tested to what extent young and older adults discriminated correctly between situations categorised as easy or difficult, and whether there was any difference in the difficulty experienced by the two groups. To this aim, we entered self-judgements in the *SOLEQ* into a cumulative link model for ordinal variables with LISTENING CONDITION (easy and difficult) as fixed effect and participants (intercept and slope) and item as random effects. This analysis revealed an effect of LISTENING CONDITION (Estimate = 3.84, *z* = 13.66, *p* < .001), indicating that participants were aware of the different degree of effort required by the two types of listening situations (easy: 1.65 ± 0.53; difficult: 3.64 ± 1.31). Note that when AGE group is also considered as a fixed effect in the model, we observed an interaction between the listening condition factor and this variable (Estimate = 4.54, *z* = 12.39, *p* < .001), revealing that the significant difference between easy and difficult for older adults is slightly smaller (Estimate = 3.18, *z* = 9.63, *p* < .001) compared to young adults (Estimate = 4.54, *z* = 12.39, *p* < .001).

Next, to investigate whether the ability to judge the listening experiences of others is influenced by one's perceptual experience, we considered as dependent variable the difference between the evaluations about one's own experience and other's experience (OSD, see 2.1.4. Analysis). We conducted two separate cumulative link models with AGE and CHARACTER of the EVALUATION (Ingroup, Outgroup) as fixed effects, and participant (intercepts and slope) and items as random effects. In the analysis of easy listening situations, we found an effect of CHARACTER of the EVALUATION (Estimate = 2.57, *z* = 5.78, *p* < .001), of AGE (Estimate = 1.08, *z* = 2.74, *p* = .006) and a significant interaction between the two factors (Estimate = 2.49, *z* = 4.16, *p* < .001) indicated that the difference between OSD values related to ingroup and outgroup was more pronounced for young adults (Estimate = 2.57, *z* = 5.78, *p* < .001) as compared to older adults (Estimate = 0.07, *z* = 0.18, *p* = .99). In other words, both groups had a smaller OSD value when considering the in-group compared to the out-group, meaning they judged the effort experiences of the in-group as more similar to their own than those of the out-group. However, the difference in OSD values between the in-group and out-group was greater among the younger participants, suggesting a greater separation between self-in-group versus self-out-group discrepancy compared to the older participants. A similar analysis of difficult listening situations revealed a significant effect of CHARACTER of the EVALUATION (Estimate = 2.20, *z* = 10.81, *p* < .001), and a significant interaction between the two factors (Estimate = 1.39, *z* = 5.12, *p* < .001) suggesting again that the difference between the self-in-group versus self-out-group discrepancy was more pronounced for young adults (Estimate = 2.20, *z* = 10.81, *p* < .001) compared to older adults (Estimate = 0.81, *z* = 4.27, *p* = .001) (see Table 1).

**Table 1.** To the right, SOLEQ mean and standard deviation values as a function of target of the evaluation; to the left, OSD mean and standard deviation values as a function of the character of evaluation (i.e., difference between Ingroup and Self and difference between Outgroup and Self). These differences represent the discrete amount of discrepancy between self and other experiences (Other-Self Discrepancy, OSD).

Age group	Target of the evaluation			Other-self discrepancy, OSD	
	Self	Ingroup	Outgroup	Ingroup - Self	Outgroup - Self
Easy					
Young	1.6 ± 0.5	1.4 ± 0.4	2.5 ± 0.9	0.38 ± 0.42	1.24 ± 0.76
Older adult	1.7 ± 0.6	1.9 ± 0.5	1.2 ± 0.2	0.47 ± 0.41	0.55 ± 0.53
Difficult					
Young	3.9 ± 1.5	4.0 ± 1.4	6.1 ± 1.4	0.91 ± 0.47	2.37 ± 1.20
Older adult	3.4 ± 1.1	3.9 ± 1.2	2.5 ± 0.9	0.78 ± 0.56	1.20 ± 0.74

To further examine the directionality of the differences between evaluations of one's own experience and others’ experiences, we conducted separate analyses for Self versus Ingroup and Self versus Outgroup judgements.

*Self versus Ingroup.* We studied judgement as a function of AGE and TARGET of the EVALUATION (Self, Ingroup) using two separate cumulative link models for easy and difficult listening conditions considering participants (intercept and slope) and item as random factors. For easy situations, we found that the response varied as a function of TARGET of the EVALUATION (Estimate = 1.91, *z* = 4.12, *p* < .001) specifically for the older adults as revealed by the interaction between the two fixed effects (Estimate = 0.90, *z* = 2.60, *p* = .009). Older adults judged their Ingroup older character to experience more effort than them (Estimate = 0.65, *z* = 2.97, *p* = .02). In contrast, young adults evaluated their Ingroup young character to experience a similar effort (Estimate = 0.25, *z* = 0.88, *p* = .82). For difficult situations, we did not find any effects (all *p values* > .11), albeit the numerical trend was similar to the results on easy situations (see means, standard deviations in Table 1).

*Self versus Outgroup.* As before, we studied judgement as a function of AGE and TARGET of the EVALUATION (Self, Outgroup) using two separate cumulative link models for easy and difficult listening conditions considering participants (intercept and slope), and item as random factors. For both easy and difficult situations we found a significant effect of AGE (easy: Estimate = 3.46, *z* = 7.39, *p* < .001; difficult: Estimate = 5.49, *z* = 9.86, *p* < .001), a significant effect of TARGET of the EVALUATION (easy: Estimate = 2.52, *z* = 7.07, *p* < .001; difficult: Estimate = 3.12, *z* = 8.66, *p* < .001), and a significant interaction between these two factors (easy: Estimate = 4.37, *z* = 8.41, *p* < .001; difficult: Estimate = 4.09, *z* = 9.60, *p* < .001). These analyses revealed that older adults perceived the young character as experiencing less effort compared to themselves (easy: Estimate = 1.85, *z* = 4.96, *p* < .001; difficult: Estimate = 1.78, *z* = 5.14, *p* < .001), while young adults perceived the older character as experiencing more effort (easy: Estimate = 2.53, *z* = 7.07, *p* < .001; difficult: Estimate = 3.12, *z* = 8.66, *p* < .001). Yet, the gap between one's own perceived effort and the perceived effort of the Outgroup was greater for young compared to older adults (see means, standard deviations in Table 1). It should also be noted that, only for the easy condition, while the self-rating of young participants did not differ from the rating provided by older adults for the young character (Estimate = 0.94, *z* = 2.00, *p* = .19), the rating given by older adults for themselves was better (they reported perceiving less effort) compared to the rating provided by young participants for the older character (Estimate = 1.62, *z* = 3.8, *p* < .001).

### Discussion

In this experiment we tested whether the inferences on the listening experiences of young and older characters are influenced by participants’ belonging to the same age group. We hypothesised that individuals would attribute listening experiences more similar to their own to the character belonging to their own age group (Ingroup) rather than to the one belonging to a different age group (Outgroup). When we asked participants to rate listening effort across various acoustic scenarios (SOLEQ), we observed that the young adults fully supported our prediction. They judged the difficulty of listening in noise of the young person (Marco) closer to their own, and instead attributed a higher listening difficulty to the older adult (Ezio). On the contrary, older adults judged their Ingroup (Ezio) to experience more effort than them in the easy listening condition. Furthermore, as expected, they also perceived the Outgroup (Marco) as experiencing less effort, although the difference compared to the Outgroup was smaller than the difference observed in the young group between themselves and the corresponding Outgroup character (see Table 1).

Coherently with the results emerging from SOLEQ, the responses provided in the IOS-pe suggested that, despite a high variability, young individuals categorised the young character as closer to themselves and the older character as farther away from themselves to a greater extent than older individuals did with the older and the younger character, respectively.

Taken together these findings may suggest that when older adults assessed their listening experience, they might not identify themselves as part of the ‘older adults’ category. Instead, they might perceive their own listening experience as generally better (i.e., less effortful) than the one of the older character. This finding contradicts our initial prediction, in which we did not anticipate differences between older and younger individuals in estimating the effort experienced by Ingroup and Outgroup characters. However, older adults’ negative judgement towards their Ingroup aligns with findings from lines of research interested in the origin of stereotypes of older adults (Harris & Associates, 1975, 1981; Schultz & Fritz, 1987). These classic studies showed that older adults rated their own lives much more positively than they rated the lives of similarly aged others. This finding was interpreted by considering two factors. On the one hand, individuals were passively exposed to information about the challenges faced by the older adults deriving from the continuous stress that mass-media put on their problems (Nemmers, 2005). This information contrasts with the subjective experience of older people and leads them to evaluate their Self more positively as compared to the general view of aging conveyed by the society. On the other hand, a non-mutual exclusive hypothesis suggests that older individuals tend to devalue the lives of their peers as a way to enhance their own self-perception, in line with the idea of the existence of a ‘self-serving bias’ (Miller & Ross, 1975).

Our results may suggest that, even in the specific context of evaluating effort during listening in noise, older individuals may rate themselves more positively (i.e., experiencing less effort) compared to similarly aged others. However, despite the fact that we specified in the description of both fictional characters that they have typical hearing, we were unable to document whether participants had erroneously represented the older character as a person with hearing deficit. Thus, to ensure that neither the young nor the older groups have implicitly associated Ezio with a representation of an older character characterised not only by a specific age but also by a certain degree of hearing deficit (e.g., due to the stereotype that older adults have age-related hearing loss), we conducted another experiment in which we introduced a new character, Gino. We specified that Gino is an older adult of the same age as Ezio, but with a mild to moderate hearing loss (different hearing experience from Ezio). We also specified that Gino's doctor would have recommended the use of hearing aids, but he decided not to use them because he thinks he can hear enough.

In this second study, *IOS-pe* and of the *SOLEQ* were administered to two new distinct samples of young and older adults. Participants were requested to estimate the difficulty experienced by themselves (Self-judgement), a young person (Marco), an older adult with normal hearing (Ezio) and an older adult with hearing deficits (Gino) in identical situations. By making explicit that Ezio is a normal-hearing older adult, we expected that older individuals’ judgements on young and normal-hearing older character would adjust and realign based on age-related logic. Therefore, if the observed effects were due to a stereotyped perception of the character Ezio, we anticipated that the presence of Gino should now lead older adults to evaluate Ezio's experience as closer to their own, while distancing Marco's experience from their own. Similarly, we expected that younger individuals would perceive Ezio as closer to themselves than before, and Marco could be positioned even closer.

## Experiment 2

### Materials and Method

#### Participants

Twenty-seven young adults (age: 25 ± 12.9, 8 males) and 27 older adults (age: 67.2 ± 4.2, 4 males) took part in Experiment 2. All participants gave their informed consent before starting the experiment, which was conducted according to the criteria of the Declaration of Helsinki (1964, amended in 2013) and in accordance with the regulations at the University of Trento. All participants reported typical hearing by self-report and did not participate in Experiment 1 (a necessary inclusion criterion).

#### Stimuli and Procedure

*Fictional characters*. Marco and Ezio were described as before. Gino was described using the following words ‘Gino is a 72-year-old individual, he is retired and has normal eyesight, but his hearing is not normal, he is hard of hearing and has a mild/moderate hearing impairment. The doctor would have recommended the use of hearing aids, but he doesn’t use them at the moment because he says he can hear enough’. Stimuli and procedure were identical to Experiment 1 with the exception that we added the new older character with hearing deficit. Thus, we required participants to rate the similarity between their acoustic perceptual experience and that of the new charter (IOS-pe) and indicated the amount of difficulty that 16 situations required for the new character (SOLEQ). No one declared to be assisted when performing the experiment. Participating required about 20 minutes. Again, we emphasised the instructions to ensure that participants focused on the character's inferred auditory perceptual experience as the key factor in reporting score for the IOS-pe.

#### Analysis

We adopted the same approach of the Experiment 1 (see section “Analysis”). We excluded two participants from the analysis on IOS-pe for lack of data (codes are ‘Lnnd’ and ‘DLZM’).

### Results

#### Judgement on the Hearing-Impaired Older Character (i.e., Gino)

**Figure 2.**
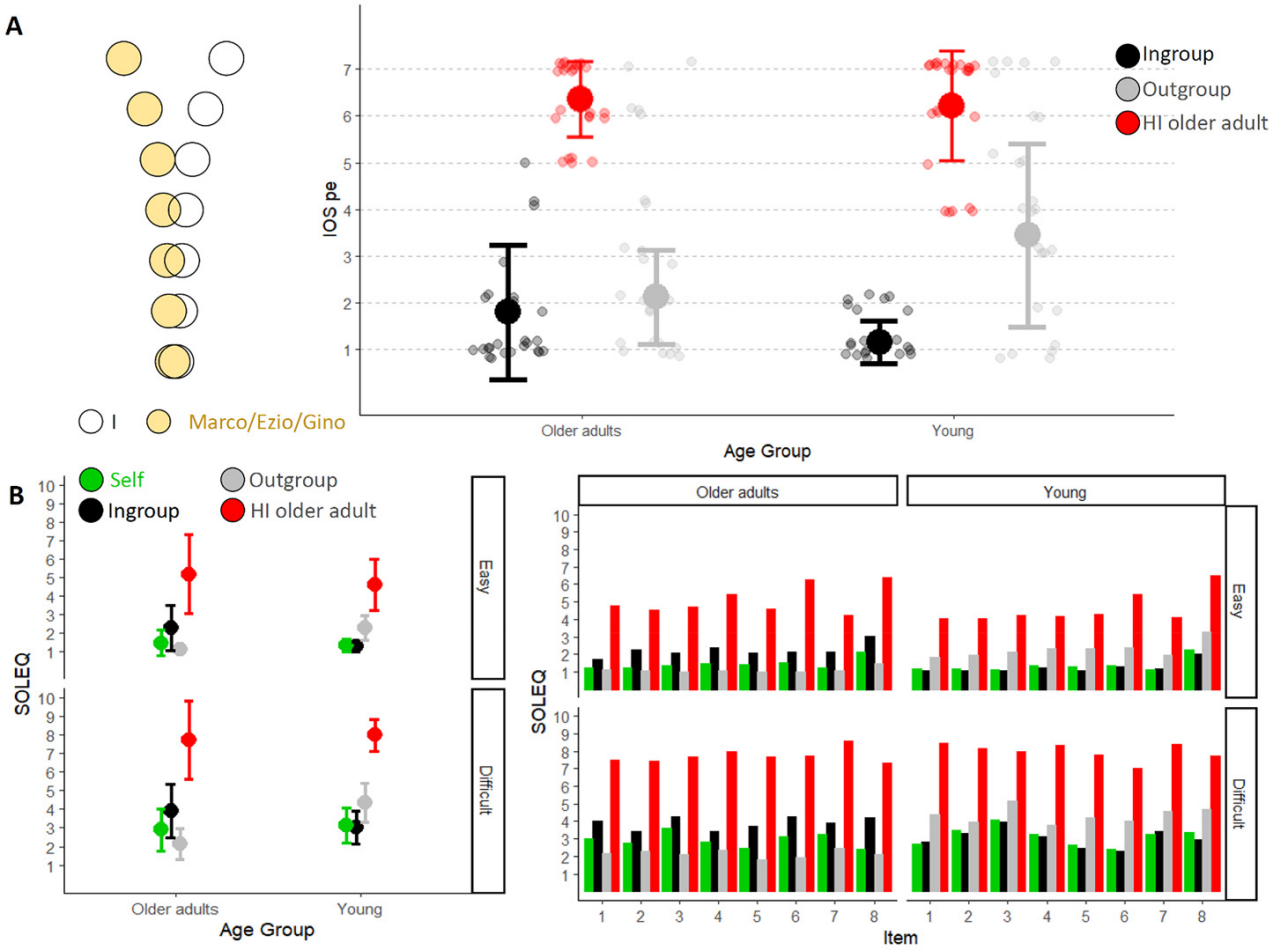
(A) On the left, an example of IOS-pe visualisation. Each circle is paired with a value solely for the purpose of selecting a response, which is then used for analysis. 1 corresponds to the most overlapping configuration, while 7 corresponds to the most distant configuration. On the right, IOS-pe mean values for young and older adults as a function of character of evaluation (Ingroup in black, Outgroup in grey, the older character with hearing impairment in red). Older adults are on the left, while young adults are on the right. The error bars represent the standard deviation, and the smaller dots represent the responses of each participant. (B) On the left, the mean response values provided by older adults (on the left) and young adults (on the right) as a function of the target of evaluation (Self in green, Ingroup in black and Outgroup in grey and the older character with hearing impairment in red). The top graph refers to the easy situations, while the bottom one refers to the difficult situations. On the right, the mean values for each individual item (8 for each condition) are shown. Again, older adults are on the left, and young adults are on the right, with the easy condition on top and the difficult condition on the bottom. The colours vary depending on the target of evaluation (green for Self, black for Ingroup, and grey for Outgroup and the older character with hearing impairment in red). See Supplementary Information for average scores.

First of all, we investigated how participants rated Gino's experience as compared to their own. Considering IOS-pe, both young and older adults rated Gino's perceptive experience as distant from their own (no one reported responses 1, 2, or 3, which corresponded to the most overlapping checks; young: 6.2 ± 1.2; older adults: 6.3 ± 0.8, Figure 2A). We entered the evaluations provided in the SOLEQ into two models considering AGE and CHARACTER of the EVALUATION as fixed effect and participants (slope and intercept) and item as random factors. Irrespective of participants’ age, and for both easy and difficult situations, participants considered hearing-impaired older character's experience more effortful as compared to their one (main effect of TARGET GROUP: Estimate = 6.05, easy: *z* = 19.52, *p* < .001; difficult: Estimate = 5.47, *z* = 20.60, *p* < .001, Figure 2B). Mean and standard deviation can be found in Table 2.

**Table 2.** To the right, SOLEQ mean and standard deviation values as a function of target of the evaluation; to the left, OSD mean and standard deviation values as a function of the character of evaluation (i.e., difference between Ingroup and Self and difference between Outgroup and Self). These differences represent the discrete amount of discrepancy between self and other experiences (Other-Self Discrepancy, OSD).

Age group	Target of the evaluation				Other-self discrepancy, OSD	
	Self	Ingroup	Outgroup	Gino	Ingroup – Self	Outgroup – Self
Easy						
Young	1.3 ± 0.2	1.3 ± 0.2	2.3 ± 0.7	4.6 ± 1.4	0.19 ± 0.19	0.94 ± 0.53
Older adult	1.5 ± 0.7	2.3 ± 1.2	1.1 ± 0.1	5.1 ± 2.1	0.83 ± 0.93	0.39 ± 0.64
Difficult						
Young	3.1 ± 0.9	3.0 ± 0.9	4.4 ± 1.0	8.0 ± 0.9	0.69 ± 0.46	1.45 ± 0.68
Older adult	2.9 ± 1.1	3.9 ± 1.5	2.1 ± 0.8	7.7 ± 2.1	1.21 ± 1.21	1.15 ± 0.98

Having established that both groups perceived Gino as distant from themselves in terms of perceptual experience and listening effort, we examined if adding Gino as context for the perceptual comparison affected the Ingroup/Outgroup judgement for the same characters considered in Experiment 1.

#### IOS-pe

We entered participants’ responses in the IOS-pe as dependent variables into a cumulative link model for ordinal variables with AGE (young, older adults) and CHARACTER of the EVALUATION (Ingroup, Outgroup) as fixed effect and participants as random effects. This analysis revealed that responses varied according to the CHARACTER of the EVALUATION (Estimate = 4.68, *z* = 4.89, *p* < .001) and AGE group (Estimate = 1.98, *z* = 2.27, *p* = .02). Both groups of participants related more with the Ingroup compared to the Outgroup fictional character. However, the interaction effect was significant (Estimate = −3.49, *z* = 3.33, *p* < .001), indicating that the difference between the Ingroup and Outgroup was greater for young participants (Ingroup: 1.1 ± 0.5; Outgroup: 3.4 ± 2.0, Estimate = 4.68*, z* = 4.89, *p* < .001) than for older adults (Ingroup: 1.8 ± 1.4; Outgroup: 2.1 ± 1.0, Estimate *=* 1.19, *z* = 2.01, *p* = .18), who rated the Outgroup character (i.e., Marco) as closer to their subjective experience. Adding EXPERIMENT (e1, e2) as fixed effect in the model does not reveal any effects involving EXPERIMENT (all *p values* > .09). This indicates that adding Gino did not alter *IOS-pe* responses.

#### SOLEQ

We entered Self-judgements in the *SOLEQ* into a cumulative link model for ordinal variables with LISTENING CONDITION (easy and difficult) as fixed effect and participants (intercept and slope) and item as random effects. This analysis revealed a significant effect of LISTENING CONDITION (Estimate = 4.56, *z* = 16.52, *p* < .001), indicating that participants were aware of the different degree of effort required by the two types of listening situations (easy: 1.41 ± 0.52; difficult: 3.02 ± 1.04). Note that when AGE group is also considered as a fixed effect in the model, we observe an interaction between the listening condition factor and this variable (Estimate = 0.93, *z* = 2.29, *p* = .02), revealing that the significant difference between easy and difficult for older adults is slightly smaller (Estimate = 4.11, *z* = 12.54, *p* < .001) compared to young adults (Estimate = 5.04, *z* = 14.42, *p* < .001).

Next, we conducted linear mixed effect models with AGE and CHARACTER of the EVALUATION (Ingroup, Outgroup) as fixed effects, and participants (intercept and slope) and item as random effects on OSD. When considering easy listening situations, this analysis revealed a significant effect of CHARACTER of the EVALUATION (Estimate = 1.64, *z* = 2.92, *p* = .003) as well as of AGE group (Estimate = 2.57, *z* = 4.02, *p* < .001). The significant interaction between these two fixed effects (Estimate = 4.91, *z* = 6.10, *p* < .001) indicated that the difference between Ingroup and Outgroup was more pronounced among young adults (Estimate = 3.26, *z* = 5.63, *p* < .001) compared to older adults (Estimate = 1.65, *z* = 2.92, *p* = .02). A similar analysis considering difficult listening situations revealed an interaction between these two effects (Estimate = 1.65, *z* = 2.59, *p* = .009), suggested that the difference between OSD vales, with lower OSD values for the Ingroup compared to the Outgroup was greater for young adults (Estimate = 1.70, *z* = 3.79, *p* < .001) compared to older adults (Estimate = 0.04, *z* = 0.10, *p* = .99).

To further examine the direction of these differences between evaluations of one's own experience and others’ experiences, we conducted separate analyses for Self versus Ingroup and Self versus Outgroup judgements.

*Self versus Ingroup.* We studied judgement as a function of AGE and TARGET of the EVALUATION (Self, Ingroup) using two separate cumulative link models for easy and difficult listening conditions considering participants (intercept and slope), and item as random factors. For easy situations, we found that response varied as a function of TARGET of the EVALUATION (Estimate = 2.75, *z* = 6.88, *p* < .001). As revealed by the interaction between the two fixed effects (Estimate = 3.57, *z* = 6.09, *p* < .001), we observed that older adults judged their Ingroup older character to experience more effort than them (Estimate = 2.75, *z* = 6.88, *p* < .001). In contrast, young adults evaluated their Ingroup young character to experience a similar effort (Estimate = 0.82, *z* = 1.89, *p* = .23). Similarly, for difficult situations, we found that response varied as a function of TARGET of the EVALUATION (Estimate = 1.94, *z* = 5.96, *p* < .001) and AGE (Estimate = 1.58, *z* = 2.63, *p* = .008). As revealed by the interaction between the two fixed effects (Estimate = 2.22, *z* = 4.89, *p* < .001), we observed that older adults judged their Ingroup older character to experience more effort than them (Estimate = 1.94, *z* = 5.96, *p* < .001). In contrast, young adults evaluated their Ingroup young character to experience a similar effort (Estimate = 0.28, *z* = 0.88, *p* = .82) (Figure 2B).

*Self versus Outgroup.* As before, we studied judgement as a function of AGE and TARGET of the EVALUATION (Self, Outgroup) using two separate cumulative link models for easy and difficult listening conditions considering participants (intercept and slope), and item as random factors. For both listening situations, we found a significant effect of AGE (easy: Estimate = 5.66, *z* = 8.83, *p* < .001; difficult: Estimate = 5.09, *z* = 8.22, *p* < .001) and of TARGET GROUP (easy: Estimate = 2.61, *z* = 4.95, *p* < .001; difficult: Estimate = 2.10, *z* = 4.58, *p* < .001) and a significant interaction between these two factors (easy: Estimate = 6.10, *z* = 8.84, *p* < .001; difficult: Estimate = 4.40, *z* = 6.87, *p* < .001). Older adults perceived the young character as experiencing less effort than themself (easy: Estimate = 2.61, *z* = 4.95, *p* < .001; difficult: Estimate = 2.10, *z* = 4.58, *p* < .001), while young adults perceived the older character as experiencing more effort (easy: Estimate = 3.49, *z* = 8.12, *p* < .001; difficult: Estimate = 2.31, *z* = 5.28, *p* < .001). Yet, the gap between one's own perceived effort and the perceived effort of the Outgroup was greater for young compared to older adults (see means and standard deviations in Table 2) (Figure 2B).

#### Self-Other Listening Experience Questionnaire (SOLEQ): Comparison Between Experiments

Note that in this section, we focused only on the effects involving EXPERIMENT.

*Self-judgement.* We entered participants’ responses about Self in two separated cumulative link models with AGE (young, older adults) and EXPERIMENT (e1, e2) as fixed effect and subject and item as random effects. No main effect or interactions involving EXPERIMENT were found in both easy (all *p values* > .33) and difficult conditions (all *p values* > .09).

*Ingroup-judgement.* Considering evaluating Ingroup listening experience, we did not find any effects involving EXPERIMENT when considering easy situations (all *p values* *>* *.15*). In contrast, when considering difficult situations, we found an effect of EXPERIMENT (Estimate = 1.66, *z* = 2.86, *p* = .004) and a significant interaction between AGE group and EXPERIMENT (Estimate = 1.68, *z* = 2.05, *p* = .04) suggesting that when evaluating Outgroup character, the responses were lower in Experiment 2 as compared to Experiment 1 only for young adults (Estimate = 1.66, *z* = 2.86, *p* = .02), but not for older adults (Estimate = 0.01, *z* = 0.03, *p* = 1.00).

*Outgroup-judgement.* Considering evaluating Outgroup listening experience, we did not find any effect of EXPERIMENT when considering easy situations (all *p values* *>* *.11*). In contrast, when considering difficult situation, we found a significant effect of AGE group (Estimate = 6.22, *z* = 10.15, *p* < .001) and of EXPERIMENT (Estimate = 2.44, *z* = 4.26, *p* < .001) suggesting that when evaluating Outgroup character, the responses were lower in Experiment 2. The interaction was only marginally significant (Estimate = 0.81, *z* = 1.69, *p* = .09), but it suggests that compared to Experiment 1, only young adults (Estimate = 2.44, *z* = 4.27, *p* < .001) changed their responses, but not older adults (Estimate = 1.07, *z* = 1.86, *p* = .24).

These results suggest that only young adults recalibrate their evaluations with respect to the Ingroup and the Outgroup with Gino's presence.

### Discussion

The results of Experiment 2 may suggest a replication of the findings from Experiment 1. Specifically, considering the results of the IOS-pe, we found an even more extreme result compared to what was observed in Experiment 1: older adults seemed to perceive their acoustic experiences similar to that of both young and older characters. Analysing the results from the SOLEQ questionnaire, we found that older adults reported perceiving less effort compared to characters of similar age, both in easy (as observed in Experiment 1) and difficult conditions (which emerged only in Experiment 2). These findings may corroborate the hypothesis that older adults do not necessarily perceive themselves as part of the ‘older adults’ group when judging their auditory experiences. Although introducing the older character with hearing impairment (i.e., Gino) led to a general recalibration in the judgements provided by the young adults, this did not alter the way in which older adults evaluated their listening experiences with respect to both Ingroup and Outgroup characters. Replicating these results in the second experiment suggest that this effect was not due to a misunderstanding in judging the older normal-hearing character.

When considering both studies presented in this article, it appears that the younger group tends to perceive older adults as individuals who generally struggle more in noisy listening situations. Moreover, older individuals with hearing impairments are perceived as facing even greater difficulties. Interestingly, even though older adults are able to represent that the younger individuals experience less effort when listening in noise compared to older adults, they seem to slightly dissociate themselves from this perception when evaluating their own effort. This finding aligns with observations dating back to the 1970s and 1980s (Harris & Associates, 1975, 1981; Schultz & Fritz, 1987) regarding older individuals’ general perspective on life. Our results may support the idea of older adults tending to evaluate their own perceptual and life experiences more positively than those of their peers.

## General Discussion

In this study, we sought to examine metacognitive perspective taking of sensory abilities in the auditory domain, by studying how younger and older adults attribute listening experiences to fictional others of the same versus different age. Across two experiments, our findings suggested that older adults rated their own acoustic experience to be better than that of fictional characters of the same age. Specifically, they judged their own perceptual experience as similar to that of the younger character and they reported perceiving less effort compared to characters of similar age.

Research on listening metacognition for older adults is scarce. While some studies have indicated weaknesses in metacognitive skills related to memory in older compared to younger individuals (Tullis & Benjamin, 2012), the influence of aging on listening metacognition remains to be determined (also see Dunlosky & Rawson, 2009). Two studies have recently addressed metacognition when listening in noisy. Giovanelli et al. (2023) showed that both young and older individuals show similar metacognitive monitoring abilities in tasks involving sentence identification in noise. In addition, they examined general knowledge for listening in noise (i.e., metacognitive knowledge), the extent to which individuals believe it is in their possibility to act (or not) on listening difficulties (i.e., internal vs. external locus of control; e.g., ‘I can act to intervene on my listening difficulties’ vs. ‘there is nothing I can do as listening difficulties are inevitable’) and opinions on the extent to which they can actually act upon listening difficulties when they can (i.e., perceived self-efficacy). In all these measures, no difference between younger and older adults with typical hearing emerged. Similarly, no differences between age groups emerged in a study examining real-time tracking of changes in perceived effort and confidence while listening to stories embedded in noise (Valzolgher et al., 2024). Participants were asked to continuously monitor their feeling of effort and confidence while listening to 3-minute-long stories in noise changing in volume at variable intervals of 10–25 seconds, resulting in changes in signal-to-noise ratio. By collecting their responses, the authors were able to extract tracks of participants’ perceived effort and confidence and tested whether they fluctuated as a function of changes in SNR. Both groups were able to modulate their reports effectively on the basis of noise fluctuation suggesting comparable metacognitive listening between young and older adults. Finally, a further study examined the ability to recognise the benefits of lip reading in noisy listening tasks and found no differences between young and older individuals, which were equally aware of this advantage (Giovanelli et al., 2025). Specifically, the authors tested participants by using virtual reality and asked them to listen to a virtual avatar, whose visibility was manipulated by changing the opacity of a panel in front of them. As conditions switched between different lip-visibility settings, measures of actual audio-visual improvements in listening were collected, along with estimates of perceived audio-visual improvement. Both older and younger adults showed comparable audio-visual improvements in the task, suggesting that age did not impact their ability to effectively exploit audio-visual integration to ease listening. Moreover, older adults demonstrated a coherent increase in their confidence levels, suggesting older adults’ preserved ability in recognising lip-reading gains while listening even beyond mere performance. Other studies (Rogers et al., 2012) have analysed metacognitive listening by considering the phenomenon of false hearing. These studies asked participants to perform a hearing in noise task in which they had to identify words presented in noise which could be congruent, incongruent, or neutral with respect to the words they learned in a prior training phase. Compared to young people, older participants relied much more on the semantic plausibility of sentences and were sure of having heard certain words, even when wrong, when they are congruent with the meaning of the sentence.

While studies focusing primarily on the perceptual aspect of listening in noise suggest preserved metacognitive abilities in older individuals, challenges seem to arise for older adults when higher-order processes are required. For instance, the studies on false hearing, which imply a semantic interpretation of sentences, and our current study, where participants were asked to build a representation of others listening experience and comparing oneself with others, involve high order top-down processes. These mechanisms may reflect the involvement of a domain-general metacognition. Conversely, tasks focusing on the perceptual aspects of listening experience may trigger more domain-specific patterns of activity. It is important to note that both general and specific forms of metacognition are present, and our assertion here is that the nature of the task can influence the extent to which these mechanisms are involved (Morales et al., 2018).

Although the present research has provided valuable insights into the role of metacognitive auditory perspective taking in young and older adults, further investigations are needed to confirm the findings observed in these two experiments while addressing some of the study's methodological limitations. Specifically, conducting the study online could have weakened the generalisability of the data because older adults who decided to take part in the experiment might have been more motivated than the average older adult and prevented us from obtaining traditional audiometric measurements, as we only collected subjective evaluations of auditory abilities. Thus, this could have an impact on participants’ responses. Furthermore, future studies could utilise measurements that more directly ask participants to describe the perceptual experience of others. For example, future studies could ask participants to perform a listening task in noise, while imagining they are listening with the ears of someone their own age or a different age. This approach would not only ask participants to provide an effort rating for an abstract situation but also in relation to a concrete, immediately experienced task. By utilising such settings, objective measures of listening performance in noise, lacking in the present study, could also be collected. In our study, we described the participants by providing elements that could help represent their auditory experience (e.g., age, hearing loss). However, other aspects, such as life circumstances and gender, were less controlled. Therefore, future studies could also consider and control these factors.

In conclusion, the results of the present study contribute to a still limited body of literature regarding metacognitive abilities related to listening in older adults. These data suggest the possible presence of difficulties in metacognitive listening for older adults, a phenomenon that has been observed to some extent in previous studies but requires further investigation in future research. The suggestion of a possible *self-serving bias* in this population may indicate that their difference in metacognitive listening is related to the relational nature of the task employed here, an aspect that has not been explored in previous research (e.g., Forsyth, 2008; Giovanelli et al., 2023; Sedikides et al., 1998). This could be interpreted as a potential effect of stereotypes on the representations that older adults have of themselves and aging (see Hausknecht et al., 2020; Meisner, 2012). This weakness in metacognitive perceptual perspective taking may be relevant to the everyday life of older adults. Since metacognition is linked to self-regulation strategies (e.g., Amran et al., 2021) and learning (e.g., Rahimi & Katal, 2012), it could potentially impact communication with younger generations. However, further studies are needed to explore this link more deeply. Future researchers could investigate whether young people, who are aware of generational differences in listening effort (as suggested in the present study), might adapt their communication style to accommodate the listening experiences of others. On the other hand, older adults who tend to underestimate their own listening effort (as suggested in the present study) may not engage in effective behaviours or adjust their communication styles, which could limit their ability to cope in noisy environments.

## Supplemental Material

sj-pdf-1-ipe-10.1177_20416695251349735 - Supplemental material for Someone else's ears: Metacognitive auditory perspective taking in young and older adults Supplemental material, sj-pdf-1-ipe-10.1177_20416695251349735 for Someone else's ears: Metacognitive auditory perspective taking in young and older adults by Chiara Valzolgher, Elena Gessa, Elena Giovanelli, and Francesco Pavani in i-Perception
