# Supplementary material for: Someone else's ears: Metacognitive auditory perspective taking in young and older adults
Source: Iperception. 2025 Jul 9;16(4):20416695251349735. doi: 10.1177/20416695251349735 (PMC12281472; doi:10.1177/20416695251349735)
Supplement: sj-pdf-1-ipe-10.1177_20416695251349735 - Supplemental material for Someone else's ears: Metacognitive auditory perspective taking in young and older adults [file sj-pdf-1-ipe-10.1177_20416695251349735.pdf]

Supplementary Information - SOLEQ

Item	Easy	Difficult
1	Quanto pensi sia difficile per XXX comprendere una conversazione uno ad uno/con un'altra persona in un luogo tranquillo?<br><br>*How difficult do you think it is for XXX to understand a one-on-one conversation with another person in a quiet place?*	Quanto pensi sia difficile per XXX comprendere una conversazione uno ad uno/con un'altra persona in un luogo tranquillo quando non riesce a vedere il viso di chi parla?<br><br>*How difficult do you think it is for XXX to understand a one-on-one conversation with another person in a quiet place when they cannot see the speaker's face?*
2	Quanto pensi sia difficile per XXX comprendere ciò che viene detto in una conversazione quando è pronunciato da un uomo?<br><br>*How difficult do you think it is for XXX to understand a conversation when it is spoken by a man?*	Quanto pensi sia difficile per XXX comprendere una conversazione al cellulare mentre c'è un rumore di sottofondo?<br><br>*How difficult do you think it is for XXX to understand a conversation on the phone while there is background noise?*
3	Quanto pensi sia difficile per XXX comprendere ciò che viene detto in una conversazione quando è pronunciato da una donna?<br><br>*How difficult do you think it is for XXX to understand a conversation when it is spoken by a woman?*	Quanto pensi sia difficile per XXX comprendere un annuncio emesso da un altoparlante quando ti trovi in un ambiente rumoroso, come per esempio un evento sportivo?<br><br>*How difficult do you think it is for XXX to understand an announcement made through a loudspeaker when you are in a noisy environment, such as a sports event?*
4	Quanto pensi sia difficile per XXX comprendere una conversazione uno ad uno/con un'altra persona mentre è ad una visita medica?<br><br>*How difficult do you think it is for XXX to understand a one-on-one conversation with another person while at a doctor's visit?*	Quanto pensi sia difficile per XXX comprendere conversazioni di gruppo quando c'è rumore di sottofondo?<br>*How difficult do you think it is for XXX to understand group conversations when there is background noise?*
5	Quanto pensi sia difficile per XXX comprendere una lezione/conferenza in una stanza piccola e tranquilla?<br><br>*How difficult do you think it is for XXX to understand a lesson or lecture in a small, quiet room?*	Quanto pensi sia difficile per XXX comprendere una conversazione uno ad uno/con un'altra persona quando la persona con cui parla sta lavando i piatti e gli dai le spalle?<br><br>*How difficult do you think it is for XXX to understand a one-on-one conversation with another person when the person they are talking to is washing dishes and has their back turned?*
6	Quanto pensi sia difficile per XXX comprendere ciò che viene detto in TV?<br><br>*How difficult do you think it is for XXX to understand what is being said on TV?*	Quanto pensi sia difficile per XXX comprendere una conversazione uno ad uno/con un'altra persona quando c'è un rumore di fondo continuo, come quello di un ventilatore<br><br>*How difficult do you think it is for XXX to understand a one-on-one conversation with another person when there is a continuous background noise, like a fan?*
7	Quanto pensi sia difficile per XXX comprendere ciò che viene detto in una conversazione quando è pronunciato da una persona che conosce bene, come un amico stretto o un membro della sua famiglia?<br><br>*How difficult do you think it is for XXX to understand a conversation when it is spoken by someone they know well, like a close friend or a family member?*	Quanto pensi sia difficile per XXX comprendere una conversazione uno ad uno/con un'altra persona quando la persona con cui parla si trova in un'altra zona/stanza della casa?<br><br>*How difficult do you think it is for XXX to understand a one-on-one conversation with another person when the person they are talking to is in a different part/room of the house?*
8	Quanto pensi sia difficile per XXX comprendere una conversazione uno ad uno/con un'altra persona in un luogo tranquillo quando non riesce a vedere il viso di chi parla?<br><br>*How difficult do you think it is for XXX to understand a one-on-one conversation with another person in a quiet place when they cannot see the speaker's face?*	Quanto pensi sia difficile per XXX comprendere una conversazione uno ad uno/con un'altra persona quando sono in corso più conversazioni contemporaneamente?<br><br>*How difficult do you think it is for XXX to understand a one-on-one conversation with another person when multiple conversations are happening simultaneously?*

Original sentences from the SOLEQ and their translation (in italics). XXX have been replaced with *yourself*, *Marco*, *Ezio*, or *Gino* as a function of the condition.

	**Experiment 1**						**Experiment 2**							
	**Young**			**Older adults**			**Young**				**Older adults**			
Item	Self	Ingroup	Outgroup	Self	Ingroup	Outgroup	Self	Ingroup	Outgroup	HI Older adult	Self	Ingroup	Outgroup	HI Older adult
Easy														
1	1.28±0.25	1.12±0.14	2.32±0.46	1.35±0.25	1.38±0.26	1.04±0.08	1.15±0.14	1.07±0.11	1.85±0.26	4.04±0.65	1.26±0.32	1.74±0.53	4.41±0.59	8.44±0.53
2	2.48±1.05	1.12±0.14	1.92±0.44	1.66±0.32	1.81±0.30	1.08±0.11	1.15±0.14	1.07±0.11	1.96±0.30	4.07±0.72	1.26±0.28	2.22±0.66	3.96±0.53	8.19±0.54
3	1.16±0.15	1.20±0.21	1.92±0.44	1.43±0.26	1.69±0.30	1.04±0.08	1.11±0.13	1.07±0.11	2.11±0.40	4.22±0.74	1.33±0.29	2.07±0.54	5.19±0.73	8.00±0.44
4	1.80±0.61	1.80±0.46	2.92±0.75	1.77±0.35	2.42±0.43	1.38±0.28	1.37±0.22	1.26±0.18	2.30±0.36	4.19±0.65	1.44±0.28	2.37±0.64	3.81±0.43	8.33±0.52
5	1.24±0.22	1.16±0.20	2.32±0.63	1.54±0.26	1.69±0.22	1.08±0.11	1.30±0.18	1.07±0.11	2.33±0.44	4.30±0.57	1.41±0.35	2.07±0.54	4.22±0.49	7.81±0.61
6	1.28±0.19	1.32±0.23	3.00±0.67	2.23±.043	2.54±0.57	1.31±0.22	1.33±0.25	1.30±0.21	2.37±0.37	5.44±0.66	1.56±0.39	2.15±0.39	4.04±0.52	7.04±0.61
7	1.16±0.20	1.20±0.17	2.12±0.48	1.38±0.26	1.50±0.26	1.12±0.13	1.11±0.13	1.15±0.14	1.96±0.36	4.11±0.76	1.26±0.26	2.11±0.53	4.56±0.56	8.41±0.65
8	2.40±0.58	2.32±0.49	3.60±0.78	2.19±0.38	2.42±0.46	1.88±0.31	2.26±0.40	2.04±0.34	3.30±0.57	6.48±0.74	2.15±0.36	3.04±0.64	4.67±0.58	7.74±0.57
Difficult														
1	3.84±0.91	4.16±0.80	6.48±0.92	3.62±0.66	3.81±0.63	2.19±0.47	2.70±0.56	2.85±0.55	1.11±0.23	4.78±1.14	3.00±0.68	4.04±0.78	2.19±0.57	7.48±1.06
2	3.80±0.88	4.12±0.76	6.04±0.83	3.00±0.44	3.35±0.60	2.35±0.39	3.48±0.40	3.33±0.54	1.04±0.08	4.56±0.93	2.78±0.46	3.44±0.83	2.30±0.54	7.44±0.92
3	4.96±0.90	4.96±0.86	7.12±0.77	3.88±0.74	4.00±0.63	2.46±0.51	4.07±0.61	3.96±0.69	1.00±0.00	4.70±0.92	3.59±0.74	4.30±0.77	2.11±0.40	7.70±1.02
4	4.32±0.81	4.00±0.64	6.20±0.79	3.31±0.54	3.92±0.56	2.62±0.44	3.26±0.64	3.15±0.43	1.07±0.11	5.44±0.94	2.81±0.58	3.44±0.47	2.33±0.41	7.96±0.88
5	3.20±0.72	3.32±0.63	5.64±0.78	3.08±0.44	4.04±0.65	2.46±0.43	2.63±0.33	2.44±0.35	1.00±0.00	4.59±0.98	2.48±0.37	3.74±0.59	1.81±0.31	7.67±0.93
6	2.52±0.66	2.56±0.63	4.68±0.77	3.15±0.62	3.50±0.67	2.04±0.37	2.41±0.37	2.30±0.29	1.00±0.00	6.26±0.93	3.15±0.57	4.30±0.66	1.93±0.29	7.74±0.84
7	4.24±0.76	4.52±0.73	6.36±0.72	4.12±0.75	4.35±0.70	3.04±0.62	3.26±0.54	3.41±0.73	1.07±0.11	4.26±0.95	3.22±0.60	3.93±0.66	2.48±0.48	8.59±0.79
8	4.12±0.84	4.56±0.73	6.64±0.81	3.15±0.63	4.00±0.57	2.50±0.40	3.37±0.54	2.93±0.42	1.48±0.23	6.41±1.04	2.41±0.47	4.19±0.83	2.11±0.51	7.33±1.00

SOLEQ average scores and 95% Confidence Intervals (CI) as a function of Experiment, Group, Character of evaluation, Listening Condition and Item.
